# Development and validation of a nomogram for predicting pulmonary complications in elderly patients undergoing thoracic surgery

**DOI:** 10.1007/s40520-024-02844-1

**Published:** 2024-10-05

**Authors:** Jingjing Liu, Dinghao Xue, Long Wang, Yanxiang Li, Luyu Liu, Guosong Liao, Jiangbei Cao, Yanhong Liu, Jingsheng Lou, Hao Li, Yongbin Yang, Weidong Mi, Qiang Fu

**Affiliations:** 1 Department of Anesthesiology, The First Medical Center, Chinese PLA General Hospital, Beijing, 100853 China; 2 Department of Anesthesiology, Beijing Tongren Hospital, Capital Medical University, Beijing, 100730 China; 3 Department of Anesthesiology, Chinese People’s Armed Police Force Hospital of Beijing, Beijing, 100027 China; 4 National Clinical Research Center for Geriatric Diseases, The Second Medical Center, Chinese PLA General Hospital, Beijing, 100853 China; 5 Department of Pain Medicine, The First Medical Center, Chinese PLA General Hospital, Beijing, 100853 China; 6 Department of Anesthesiology, The 71st Group Army Hospital of CPLA Army, Xuzhou, 221004 China; 7 Department of Anesthesiology, 947 Hospital of Chinese PLA, Kashi Prefecture, Xinjiang, 844200 China

**Keywords:** Postoperative pulmonary complications (PPCs), Elderly patients, Thoracic surgery, Nomogram, Prediction

## Abstract

**Background:**

Postoperative pulmonary complications (PPCs) remain a prevalent concern among elderly patients undergoing surgery, with a notably higher incidence observed in elderly patients undergoing thoracic surgery. This study aimed to develop a nomogram to predict the risk of PPCs in this population.

**Methods:**

A total of 2963 elderly patients who underwent thoracic surgery were enrolled and randomly divided into a training cohort (80%, *n* = 2369) or a validation cohort (20%, *n* = 593). Univariate and multivariate logistic regression analyses were conducted to identify risk factors for PPCs, and a nomogram was developed based on the findings from the training cohort. The validation cohort was used to validate the model. The predictive accuracy of the model was evaluated by receiver operating characteristic (ROC) curve, area under ROC (AUC), calibration curve, and decision curve analysis (DCA).

**Results:**

A total of 918 (31.0%) patients reported PPCs. Nine independent risk factors for PPCs were identified: preoperative presence of chronic obstructive pulmonary disease (COPD), elevated leukocyte count, higher partial pressure of arterial carbon dioxide (PaCO_2_) level, surgical site, thoracotomy, intraoperative hypotension, blood loss > 100 mL, surgery duration > 180 min, and malignant tumor. The AUC value for the training cohort was 0.739 (95% *CI*: 0.719–0.762), and it was 0.703 for the validation cohort (95% *CI*: 0.657–0.749). The *P-*values for the Hosmer-Lemeshow test were 0.633 and 0.144 for the training and validation cohorts, respectively, indicating a notable calibration curve fit. The DCA curve indicated that the nomogram could be applied clinically if the risk threshold was between 12% and 84%, which was found to be between 8% and 82% in the validation cohort.

**Conclusion:**

This study highlighted the pressing need for early detection of PPCs in elderly patients undergoing thoracic surgery. The nomogram exhibited promising predictive efficacy for PPCs in elderly patients undergoing thoracic surgery, enabling the identification of high-risk patients and consequently aiding in the implementation of preventive interventions.

**Graphical Abstract:**

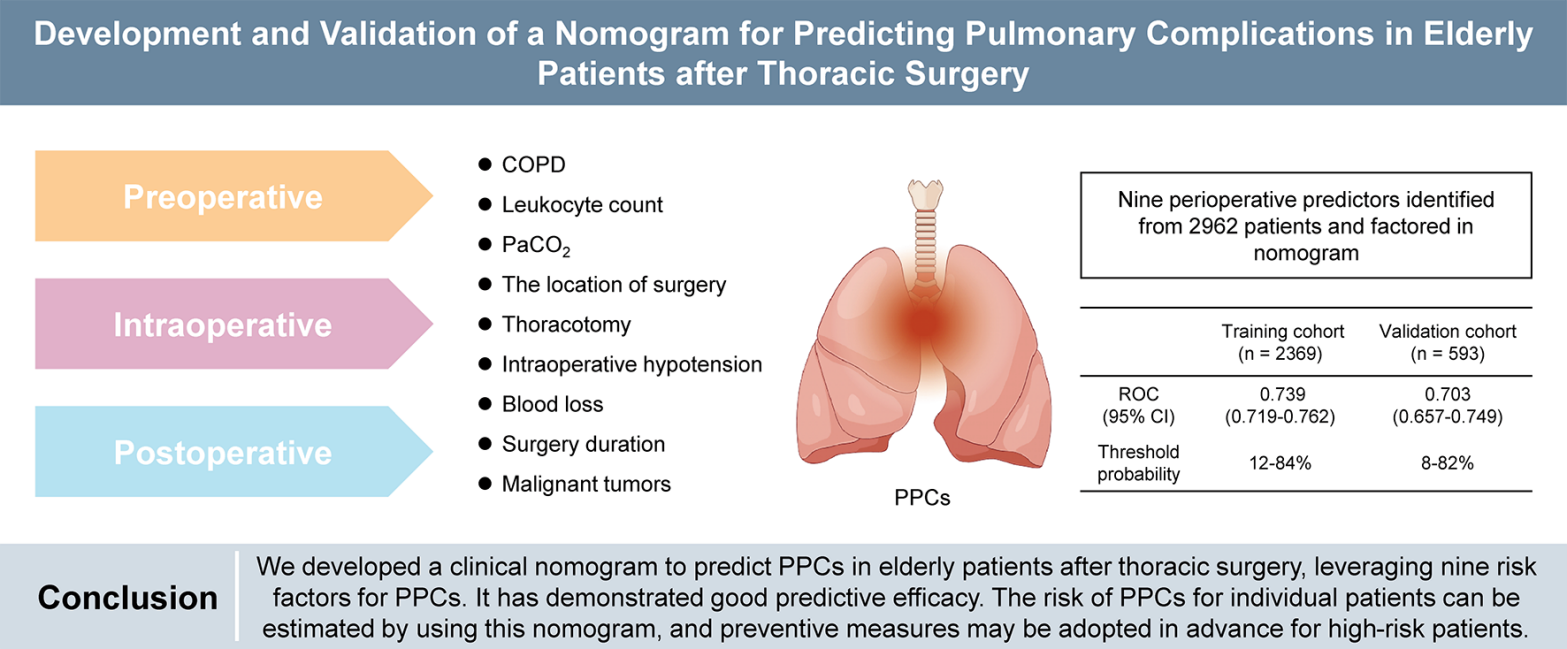

**Supplementary Information:**

The online version contains supplementary material available at 10.1007/s40520-024-02844-1.

## Introduction

Thoracic surgery is a common and indispensable modality for diagnosing and treating an array of thoracic diseases [1], including lung cancer, esophageal cancer, mediastinal masses, and other conditions affecting the chest. Thoracic surgeries typically involve intrathoracic irrigation, single-lung ventilation, and carry the risk of barotrauma, volutrauma, ventilator-induced lung injury, and acute or chronic pain [2, 3]. These unique characteristics contribute to the occurrence of numerous postoperative pulmonary complications (PPCs), including pleural effusion, pneumonia, pneumothorax, pulmonary atelectasis, respiratory failure, bronchospasm, and bronchopleural fistula [4], remarkably impacting the patient’s prognosis [5]. Studies have reported varying rates of PPCs, ranging from 20 to 70% [6–8]. One of the most significant consequences is an increased risk of mortality. Several studies have demonstrated higher mortality rates in elderly patients who develop PPCs [9, 10]. Moreover, patients who develop complications mainly experience prolonged recovery, increased pain, and decreased functional capacity. These factors can significantly impact individuals’ ability to perform daily activities, leading to their diminished quality of life [8, 9]. Additionally, pulmonary complications can result in long-term respiratory sequelae, such as impaired pulmonary function, exercise intolerance, and chronic respiratory symptoms, further exacerbating the negative impact on the patient’s overall well-being [10]. The aging population worldwide is increasing, leading to an increase in the number of elderly individuals undergoing thoracic surgery. PPCs following thoracic surgery are common, and elderly patients are particularly susceptible to these conditions [11]. The greater incidence of these complications in elderly patients can be attributed to age-related physiological changes, decreased respiratory reserve, comorbidities, and impaired immune function [4, 12]. Furthermore, the type of surgery, such as pneumonectomy or lobectomy, also influences the risk of complications, and pneumonectomy is associated with higher rates versus other procedures [13]. However, investigations specifically targeting PPCs, especially following thoracic surgery, in elderly individuals are rare.

Given the significant impact of PPCs on elderly patients, there is a need for the development of predictive models that can identify high-risk individuals. The primary objective of this study was to develop and validate a nomogram for predicting PPCs in elderly patients undergoing thoracic surgery. By identifying high-risk patients, healthcare professionals can implement targeted interventions to minimize the occurrence and severity of these complications.

## Methods

### Study design and patients’ selection

This study was reviewed and approved by the Ethics Committee of the First Medical Center of Chinese PLA General Hospital (Grant No. S2019-311-03), and the patient’s written consent was waived because the data used were anonymized and because the study was retrospective. This study was conducted in accordance with the *Transparent Reporting of a multivariable prediction model for Individual Prognosis Or Diagnosis (TRIPOD)* checklist [14]. The diagnosis of PPCs was carried out by two experienced clinicians who independently reviewed the clinical data of the patients. In cases where there was a disagreement between the two clinicians, the diagnosis was further discussed and resolved by the research team to ensure accuracy and consistency. This process was implemented to minimize subjective bias and enhance the reliability of the PPC diagnoses.

The basic and clinical data of 3079 elderly patients who underwent thoracic surgery in the Thoracic Perioperative Database for Geriatrics in the First Medical Center of Chinese PLA General Hospital (Beijing, China) from January 2012 to August 2019 were retrospectively analyzed.

**Fig. 1:**
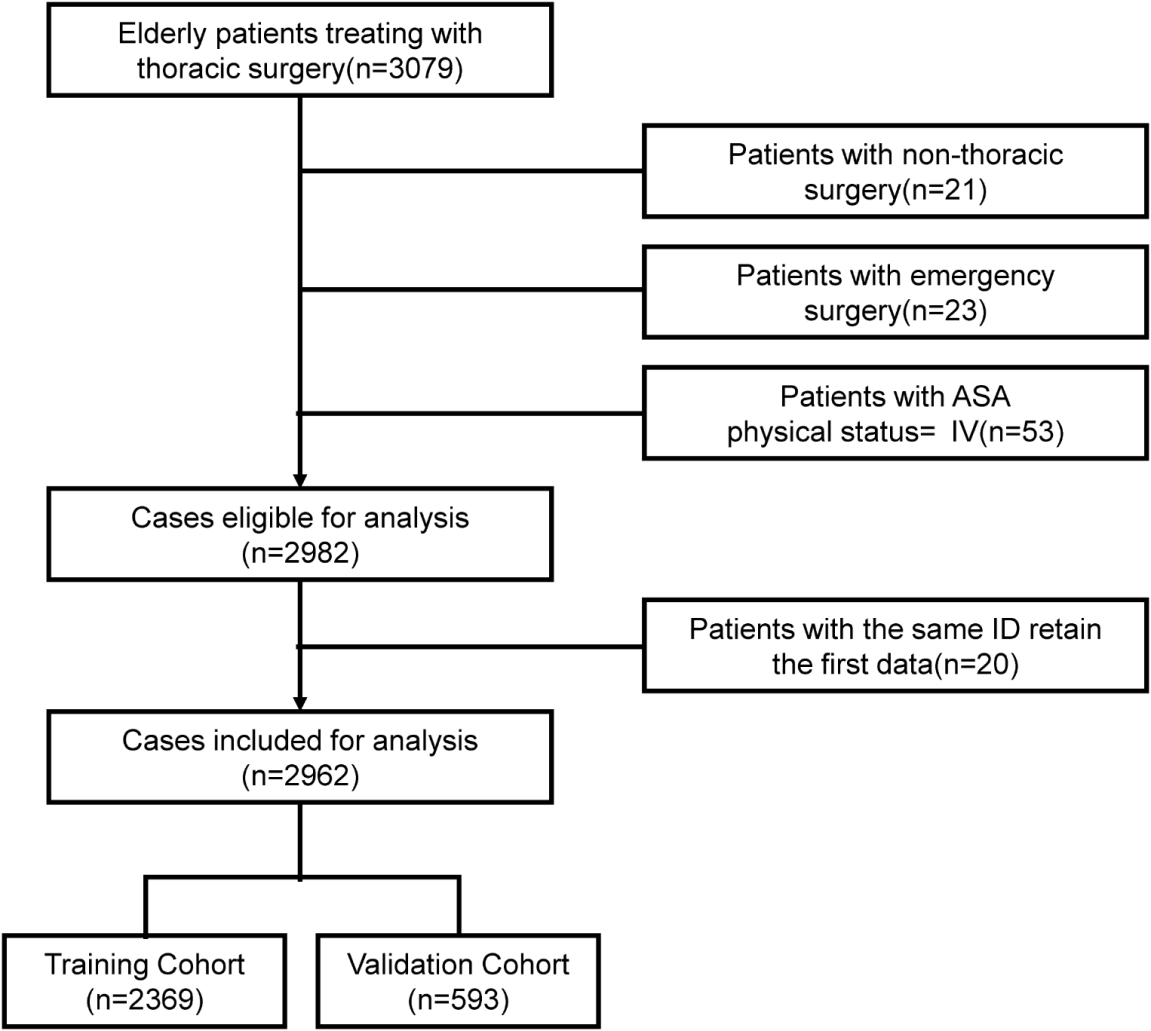
Details on study recruitment

The study population consisted of elderly patients who aged ≥ 65 years and underwent thoracic surgery under general anesthesia at the First Medical Center of the Chinese PLA General Hospital. The types of thoracic surgeries included were not limited to specific diseases, while involved a range of conditions requiring such surgical intervention. These conditions included both cancerous (e.g., lung and esophageal cancer) and non-cancerous diseases. The inclusion criteria were summarized as follows: (1) Patients who aged ≥ 65 years; (2) Patients who underwent thoracic surgery under general anesthesia at the specified hospital. The exclusion criteria were summarized as follows: (1) Undergoing non-thoracic surgery; (2) Undergoing emergency surgery; (3) Patients’ American Society of Anesthesiologists (ASA) physical status was ≥ IV; (4) The same ID was used to retain the first data entry only. The term “thoracic surgery” in this context is comprehensive, encompassing surgeries for both oncologic conditions (such as lung and esophageal benign/malignant tumors) and other thoracic conditions (such as thymoma, teratoma, ectopic thyroid tumor, lymphoma, etc.). This inclusion is reflective of the varied nature of thoracic surgical procedures performed in the geriatric population at our institution. Figure 1 illustrates the flowchart of patients’ selection.

All enrolled patients were randomly assigned to the training cohort or validation cohort at an 8:2 ratio using a random split-sample method. The training cohort was utilized to develop the predictive nomogram, while the validation cohort was used to verify the performance of the nomogram.

### Surgical protocols and Perioperative Care

#### Surgical approach

Patients in this study underwent various types of thoracic surgeries, including open thoracotomy, video-assisted thoracoscopic surgery (VATS), and robot-assisted surgery (RAS). The selection of surgical approach was determined by the operating surgeon based on the patient’s clinical condition, tumor location, and other relevant factors.

#### Perioperative care

Perioperative care protocols included the following components: Preoperative Care: (1) Patients received preoperative instructions on smoking and alcohol cessation at least two weeks before surgery; (2) Nutritional assessments were conducted, and perioperative nutritional therapy was provided as needed; (3) Respiratory rehabilitation exercises were taught to patients to improve lung function before surgery.

#### Intraoperative care

(1) Standardized anesthesia protocols were followed, including the use of inhalation anesthesia and thyrocricocentesis if required; (2) Intraoperative monitoring included heart rate, mean arterial pressure (MAP), and continuous assessment for arrhythmias and hypotension.

#### Postoperative care

(1) Early mobilization protocols were implemented to reduce the risk of PPCs; (2) Postoperative respiratory rehabilitation, including incentive spirometry and chest physiotherapy, was provided to enhance lung recovery; (3) Pain management was optimized using a combination of analgesics, including NSAIDs and opioids as required.

### Variables and outcomes

The following data of eligible patients were collected from the database: (1) basic characteristics, including age, sex, body mass index (BMI), smoking and drinking history, ASA physical status; (2) preoperative comorbidities, such as hypertension, diabetes, coronary heart disease, arrhythmia, cerebrovascular disease, chronic obstructive pulmonary disease (COPD), asthma, renal insufficiency; (3) preoperative laboratory tests, including leukocyte count, hemoglobin, partial pressure of arterial carbon dioxide (PaCO_2_), partial pressure of arterial oxygen (PaO_2_), serum potassium, sodium and glucose; (4) preoperative medications, involving angiotensin-converting enzyme inhibitor (ACEI) drugs, statin drugs, nonsteroidal anti-inflammatory drugs (NSAIDs), β-blockers, and calcium channel blockers; (5) preoperative evaluation of heart rate and mean artery pressure (MAP); (6) intraoperative and postoperative variables, including the usage of inhalation anesthesia, thyrocricocentesis, the occurrence of intraoperative arrhythmia and hypotension (MAP < 65 mmHg; systolic blood pressure (SBP) reduction > 20%; SBP < 100 mmHg [15]), the surgical site, the type of surgery, the infusion volume, the usage of glucocorticoids, blood loss, operation time, the usage of postoperative analgesia, and pathological results.

### PPC evaluation

According to previous studies, PPC was defined as a combination of pneumonia, pleural effusion, pneumothorax, pulmonary atelectasis, respiratory failure, and bronchospasm within 30 days after surgery.

The diagnosis of pneumonia was made based on the presence of any of the following criteria: (1) Postoperative pulmonary computed tomography (CT) or chest X-ray revealing the emergence of a new pulmonary infection, or two consecutive imaging examinations post-surgery, indicating the presence of pulmonary infection or a progressive deterioration of pneumonia; (2) The preoperative diagnosis did not include pneumonia, while upon discharge, the diagnosis was revised to include pneumonia; (3) Postoperative imaging reveals evidence of pulmonary infection (excluding postoperative chronic inflammation and minimal inflammation) in combination with any of the following: replacement of antibiotics after a minimum interval of 8 h, body temperature exceeding 38.3 °C, leucocyte count surpassing 12 × 10^9^/L, or falling below 4 × 10^9^/L; or (4) Positive sputum culture after surgery, along with any of the following criteria: replacement of antibiotics after a minimum interval of 8 h, body temperature exceeding 38.3 °C, white blood count surpassing 12 × 10^9^/L, or falling below 4 × 10^9^/L [16].

Pleural effusion: the radiographic diagnosis of the chest, as interpreted by the radiologist, suggested the presence of pleural effusion, excluding a small amount of pleural effusion [10].

Pneumothorax: air in the pleural space with no vascular bed surrounding the visceral pleura [10].

Pulmonary atelectasis: lung opacification with mediastinal shift, hilum or hemidiaphragm shift toward the affected area, with compensatory hyperinflation in the adjacent non-atelectatic lung [10].

Respiratory failure was defined as follows: postoperative PaO_2_ < 60 mmHg on room air, a PaO_2_: FIO_2_ ratio < 300 mmHg, or arterial oxyhemoglobin saturation measured with pulse oximetry < 90% and requiring oxygen therapy [10].

Bronchospasm was defined as newly identified expiratory wheezing treated with bronchodilators that appeared in medical records such as clinical course notes, consultation notes, and discharge diagnoses [9].

Two experienced clinicians diagnosed the PPCs based on the clinical data of the patients. In cases of dispute, the diagnosis was discussed by the research team [17].

### Development and evaluation of the nomogram

To determine the potential risk factors associated with PPCs in the training cohort, we compared the preoperative, intra- and postoperative basic and clinical data. All factors with *P* < 0.05 were then included in a multivariate logistic regression analysis. Subsequently, with R project software (version 4.1.1), a predictive model and nomogram were developed using all the independent risk factors. To ensure the reliability of the findings, internal validation was performed using the bootstrap method with 1000 repetitions. The discrimination efficiency of the predictive nomogram was evaluated by the receiver operating characteristic (ROC) curve and the optimal cutoff values. Furthermore, the calibration power of the model was evaluated using the Hosmer-Lemeshow test and calibration curve. These assessments aimed to determine how well the model’s predicted probabilities aligned with the observed probabilities of PPCs. Finally, the clinical utility of the predictive calibration plot was evaluated using decision curve analysis (DCA).

### Secondary analysis

To address concerns about the inclusion duration and potential variability due to evolving treatment guidelines, secondary analysis was conducted concentrating on a shorter inclusion period. Specifically, data from the most recent 5 years in our dataset were analyzed (2014 to 2019). This secondary analysis aimed to evaluate whether there would be significant differences in outcomes compared with the full 7-year inclusion period.

### Incorporation of year of surgery

Furthermore, to account for potential confounding effects due to changes in treatment protocols over time, the year of surgery was included as a variable in the multivariate analysis. By incorporating the year of surgery, we aimed to control for temporal variations and ensure that the predictive model could accurately reflect the impact of different treatments on patient outcomes.

### Statistical analysis

All the statistical analyses and data management were performed in SPSS version 26 (IBM Corporation, Armonk, NY, USA) and R Project software (v4.1.1; http://www.R-project.org) with the packages of mice, rms and rmda. The normally distributed data are reported as the mean ± standard deviation (SD). Continuous non-normal data are reported as the median and interquartile range (IQR). Categorical data are expressed as the frequency and proportion. The Student’s t-test, the Mann-Whitney U nonparametric test, the Pearson Chi-squared test, Fisher’s exact test and the continuity-adjusted chi-square test were used to compare the differences between groups. A *P* value < 0.05 indicated a statistically significant difference, and the *P* values were two-sided.

## Results

### Patients’ baseline demographic and clinical characteristics

A total of 2962 patients met the eligibility criteria between January 2012 and August 2019. Patients’ baseline demographic and clinical characteristics, particularly in patients with and without PPCs, are summarized in Supplementary Table S1. Among 2962 patients, 918 (31.0%) patients developed 1324 PPCs, whereas 300 (24.7%) patients developed multiple PPCs: 222 (24.18%) patients had 2 PPCs, 54 (5.88%) patients had 3 PPCs, and 24 (0.2%) patients had ≥ 4 PPCs. The most frequent complication was pleural effusion (636 patients, 48.04%), followed by pneumonia (416, 31.42%), pneumothorax (147, 11.10%), pulmonary atelectasis (75, 5.66%), respiratory failure (46, 3.47%), and bronchospasm (4, 0.30%).

The selected patients were randomly assigned to the training cohort (*n* = 2369) or the validation cohort (*n* = 593) at a ratio of 8:2, and all baseline demographic and clinical characteristics did not significantly differ between the two cohorts (Table 1).

**Table 1:** Patients’ basic and clinical characteristics of the training cohort and validation cohort

Variables	Total (*n* = 2962)	Training cohort (*n* = 2369)	Validation cohort (*n* = 593)	*P* value	
Age (years) (IQR)	69 (66, 72)	69 (66, 72)	69 (67, 72)	0.705	
Sex (male) (*n*, %)	1807 (61.01)	1446 (61.04)	361 (60.88)	0.943	
BMI (kg/m^2^) (IQR)	24.24 (22.32, 26.34)	24.11 (22.21, 26.22)	24.34 (22.21, 26.37)	0.814	
Smoking history (*n*, %)	1181 (39.87)	946 (39.93)	235 (39.63)	0.893	
Drinking history (*n*, %)	844 (28.49)	661 (27.90)	183 (30.86)	0.154	
ASA physical status				0.782	
I (*n*, %)	38 (1.28)	31 (1.31)	7 (1.18)		
II (*n*, %)	2505 (84.57)	1998 (84.34)	507 (85.50)		
III (*n*, %)	419 (14.15)	340 (14.35)	79 (13.32)		
Preoperative comorbidities					
Hypertension (*n*, %)	1180 (39.84)	930 (39.26)	250 (42.16)	0.197	
Diabetes (*n*, %)	620 (20.93)	489 (20.64)	131 (22.09)	0.438	
Coronary heart disease (*n*, %)	327 (11.04)	258 (10.89)	69 (11.64)	0.657	
Cardiac arrhythmia (*n*, %)	289 (9.76)	237 (10.00)	52 (8.77)	0.407	
Cerebrovascular disease (*n*, %)	332 (11.21)	266 (11.23)	66 (11.13)	0.999	
COPD (*n*, %)	547 (18.47)	438 (18.49)	109 (18.38)	0.999	
Asthma (*n*, %)	13 (0.44)	9 (0.38)	4 (0.67)	0.533	
Renal insufficiency (*n*, %)	24 (0.81)	18 (0.76)	6 (1.01)	0.722	
Preoperative laboratory tests					
Leukocyte count(×10^9^/L) (IQR)	5.95 (4.97, 7.07)	5.94 (4.96, 7.04)	6.03 (5.01, 7.22)	0.137	
Hemoglobin (g/L) (IQR)	137.56 (127.34, 147.81)	137.24 (127.86, 147.94)	137.62 (127.59, 147.20)	0.513	
PaO_2_ (mmHg) (IQR)	96.35 (95.54, 97.45)	96.29 (95.37, 97.86)	96.88 (95.76, 97.94)	0.562	
PaCO_2_ (mmHg) (IQR)	45.21 (42.21, 47.52)	44.93 (42.35, 47.43)	45.13 (42.34, 47.88)	0.624	
Potassium (mmol/L) (IQR)	4.11 (3.88, 4.32)	4.12 (3.88, 4.32)	4.18 (3.88, 4.32)	0.836	
Sodium (mmol/L) (IQR)	142.56 (140.94, 143.92)	142.51 (141.03, 143.90)	142.49 (140.92, 143.86)	0.658	
Glucose (mmol/L) (IQR)	4.98 (4.59, 5.57)	4.96 (4.60, 5.55)	4.99 (4.58, 5.61)	0.999	
Preoperative medication					
ACEI drugs (*n*, %)	90 (3.04)	73 (3.08)	17 (2.87)	0.785	
Statin drugs (*n*, %)	155 (5.23)	120 (5.07)	35 (5.90)	0.474	
NSAIDs (*n*, %)	2679 (90.45)	2139 (90.29)	540 (91.06)	0.622	
β -blocker (*n*, %)	257 (8.68)	208 (8.78)	49 (8.26)	0.750	
Calcium channel blocker (*n*, %)	698 (23.57)	545 (23.01)	153 (25.81)	0.167	
Preoperative heart rate (IQR)	74 (68, 78)	74 (68, 78)	73 (68, 78)	0.671	
Preoperative MAP (mmHg) (SD)	95.55 (10.97)	95.51 (10.90)	95.71 (11.24)	0.682	

*Abbreviations* *BMI* body mass index; *ASA* American Society of Anesthesiologists; *COPD* chronic obstructive pulmonary disease; *PaO*_*2*_ arterial partial pressure of oxygen; *PaCO*_*2*_ arterial partial pressure of carbon dioxide; *ACEI* angiotensin-converting enzyme inhibitors; *NSAIDs* non-steroidal anti-inflammatory drugs; *MAP* mean artery pressure; *IQR* interquartile range; *SD* standard deviation

### Identification of risk factors for PPCs

In the training cohort, patients were divided into PPC and non-PPC groups based on the presence of PPCs. The PPC group exhibited a significantly greater incidence of COPD (*P* < 0.001), elevated leukocyte count (*P* < 0.001), and PaCO_2_ level (*P* = 0.013). Additionally, a greater proportion of patients in the PPC group experienced intraoperative hypotension (*P* < 0.001), underwent esophageal surgery (*P* < 0.001) and thoracotomy (*P* < 0.001), had greater blood loss (*P* < 0.001), a longer operation time (*P* < 0.001), and tended to have a malignant tumor (*P* < 0.001) (Tables 2 and 3).

**Table 2:** Basic and clinical characteristics of patients with or without PPCs in the training cohort

Variables	Total (*n* = 2369)	PPCs group (*n* = 747)	Non-PPCs group (*n* = 1622)	*P* value	
Age (years) (IQR)	69 (66, 72)	69 (66, 73)	69 (66, 72)	0.355	
Sex (male) (*n*, %)	1446 (61.04)	469 (62.78)	977 (60.23)	0.237	
BMI (kg/m^2^) (IQR)	24.11 (22.21, 26.92)	24.44 (22.49, 26.82)	24.25 (22.31, 26.30)	0.296	
Smoking history (*n*, %)	946 (39.93)	310 (41.50)	636 (39.21)	0.291	
Drinking history (*n*, %)	661 (27.90)	228 (30.52)	433 (26.70)	0.054	
ASA physical status				0.687	
I (*n*, %)	31 (1.31)	12 (1.61)	19 (1.17)		
II (*n*, %)	1998 (84.34)	628 (84.07)	1370 (84.46)		
III (*n*, %)	340 (14.35)	107 (14.32)	233 (14.36)		
Preoperative comorbidities					
Hypertension (*n*, %)	930 (39.26)	280 (37.48)	650 (40.07)	0.230	
Diabetes (*n*, %)	489 (20.64)	159 (21.29)	330 (20.35)	0.599	
Coronary heart disease (*n*, %)	258 (10.89)	74 (9.91)	184 (11.34)	0.331	
Arrhythmia (*n*, %)	237 (10.00)	71 (9.50)	166 (10.23)	0.634	
Cerebrovascular disease (*n*, %)	266 (11.23)	80 (10.71)	186 (11.47)	0.636	
COPD (*n*, %)	438 (18.49)	175 (23.43)	263 (16.21)	**< 0.001**	
Asthma (*n*, %)	9 (0.38)	2 (0.27)	7 (0.43)	0.808	
Renal insufficiency (*n*, %)	18 (0.76)	4 (0.54)	14 (0.86)	0.393	
Preoperative laboratory tests					
Leukocyte count (×10^9^/L) (IQR)	5.94 (4.96, 7.04)	6.13 (5.16, 7.32)	5.85 (4.86, 6.91)	**< 0.001**	
Hemoglobin (g/L) (IQR)	137.24 (127.86, 147.94)	138.22 (128.69, 146.37)	137.51 (127.94, 147.11)	0.467	
PaO_2_ (mmHg) (IQR)	96.29 (95.37, 97.86)	96.13 (95.59, 97.35)	96.48 (95.88, 97.62)	0.624	
PaCO_2_ (mmHg) (IQR)	44.9 (42.3, 47.4)	45.6 (43.2, 47.7)	44.8 (42.3, 47.3)	**0.013**	
Potassium (mmol/L) (IQR)	4.12 (3.88, 4.32)	4.12 (3.92, 4.34)	4.09 (3.87, 4.32)	0.158	
Sodium (mmol/L) (IQR)	142.51 (141.03, 143.90)	142.32 (141.67, 143.63)	142.65 (141.14, 143.48)	0.224	
Glucose (mmol/L) (IQR)	4.96 (4.60, 5.55)	4.98 (4.61, 5.545)	4.96 (4.6, 5.56)	0.935	
Preoperative medication					
ACEI drugs (*n*, %)	68 (2.87)	28 (3.75)	40 (2.47)	0.109	
Statin drugs (*n*, %)	120 (5.07)	32 (4.28)	88 (5.43)	0.282	
NSAIDs (*n*, %)	2139 (90.29)	674 (90.23)	1465 (90.32)	0.999	
β -blocker (*n*, %)	208 (8.78)	67 (8.97)	141 (8.69)	0.887	
Calcium channel blocker (*n*, %)	545 (23.00)	162 (21.67)	383 (23.61)	0.326	
Preoperative heart rate (IQR)	74 (68, 78)	74 (69, 78)	73 (68, 78)	0.093	
Preoperative MAP (mmHg) (SD)	95.51 (10.90)	95.26 (11.01)	96.08 (10.81)	0.089	

*Abbreviations* *BMI* body mass index; *ASA* American Society of Anesthesiologists; *COPD* chronic obstructive pulmonary disease; *PaO*_*2*_ arterial partial pressure of oxygen; *PaCO*_*2*_ arterial partial pressure of carbon dioxide; *ACEI* angiotensin-converting enzyme inhibitors; *NSAIDs* non-steroidal anti-inflammatory drugs; *MAP* mean artery pressure; *IQR* interquartile range; *SD* standard deviation

**Table 3:** Intra- and postoperative data of patients with or without PPCs in the training cohort

Variables	Total (*n* = 2369)	PPCs group (*n* = 747)	Non-PPCs group (*n* = 1622)	*P* value	
Inhalation anesthesia (n, %)	2272 (95.91)	715 (95.72)	1557 (95.99)	0.752	
Thyrocricocentesis (n, %)	412 (17.39)	137 (18.34)	275 (16.95)	0.408	
Intraoperative arrhythmia (n, %)	7 (0.30)	3 (0.40)	4 (0.25)	0.812	
Intraoperative hypotension (n, %)	464 (19.59)	193 (25.84)	271 (16.71)	**< 0.001**	
The location of surgery				**< 0.001**	
Lung (n, %)	1683 (71.04)	403 (53.95)	1280 (78.91)		
Esophagus (n, %)	595 (25.12)	324 (43.37)	271 (16.71)		
Mediastinum (n, %)	79 (3.33)	18 (2.41)	61 (3.76)		
Other (n, %)	12 (0.51)	2 (0.27)	10 (0.62)		
Type of surgery				**< 0.001**	
Thoracotomy (n, %)	646 (27.27)	323 (43.24)	323 (19.91)		
Thoracoscopic (n, %)	1723 (72.73)	424 (56.76)	1299 (80.09)		
Infusion volume (ml/kg·h) (IQR)	8.02 (6.44, 9.94)	8.00 (6.52, 9.88)	8.05 (6.42, 9.99)	0.920	
Usage of glucocorticoids (n, %)	1795 (75.77)	578 (77.38)	1217 (75.03)	0.216	
Blood loss				**< 0.001**	
≤ 100 ml (n, %)	1699 (71.72)	405 (54.22)	1294 (79.78)		
> 100 ml (n, %)	670 (28.28)	342 (45.78)	328 (20.22)		
Surgery duration				**< 0.001**	
≤ 180 min (n, %)	1373 (57.96)	267 (35.74)	1106 (68.19)		
> 180 min (n, %)	996 (42.04)	480 (64.26)	516 (31.81)		
Postoperative analgesia (n, %)	1878 (79.27)	603 (80.72)	1275 (78.61)	0.238	
Pathology result				**< 0.001**	
Benign (n, %)	195 (8.23)	34 (4.55)	161 (9.93)		
Malignancy (n, %)	2174 (91.77)	713 (95.45)	1461 (90.07)		

*Abbreviations* *IQR* interquartile range

Variables with *P* < 0.05 from the univariate analysis were subsequently incorporated into the multivariate logistic regression analysis. Notably, nine factors, including the preoperative presence of COPD (*P* < 0.001), an elevated leukocyte count (*P* = 0.001), a higher PaCO_2_ level (*P* = 0.013), intraoperative hypotension (*P* < 0.001), esophageal surgery (*P* < 0.001), thoracotomy (*P* < 0.001), blood loss > 100 mL (*P* < 0.001), operation time > 180 min (*P* < 0.001), and malignant tumors (*P* = 0.042), were significantly associated with an increased risk of PPCs (Table 4).

**Table 4:** Multivariate analysis of risk factors for PPCs

Variables	B	Wald	*P* value	OR	95% CI
COPD	0.467	2.543	**< 0.001**	1.595	1.260–2.543
Leukocyte count	0.097	1.213	**0.001**	1.101	1.041–1.166
PaCO_2_	0.030	1.061	**0.013**	1.030	1.006–1.055
Intraoperative hypotension	0.411	2.275	**< 0.001**	1.508	1.198–1.897
Location of surgery					
Lung (reference)					
Esophagus	0.502	2.731	**< 0.001**	1.653	1.281–2.132
Mediastinum	0.386	2.166	0.252	1.472	0.748–2.827
Other	-0.091	0.834	0.911	0.913	0.134–3.785
Thoracotomy	0.543	2.964	**< 0.001**	1.722	1.359–2.177
Blood loss > 100 mL	0.470	2.562	**< 0.001**	1.601	1.281–1.998
Surgery duration > 180 min	0.825	5.203	**< 0.001**	2.281	1.834–2.838
Malignant tumor	0.493	2.678	**0.042**	1.636	1.034–2.673

*Abbreviations* *COPD* chronic obstructive pulmonary disease; *PaCO*_*2*_ arterial partial pressure of carbon dioxide.

### Predictive performance and validation of the nomogram for PPCs

**Fig. 2:**
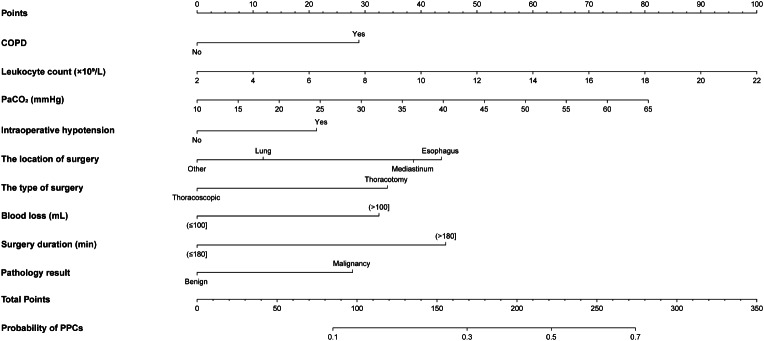
A nomogram for predicting PPCs in elderly patients after thoracic surgery. *Abbreviations* *COPD* chronic obstructive pulmonary disease; *PaCO*_*2*_ arterial partial pressure of carbon dioxide; *PPCs* postoperative pulmonary complications

According to the coefficients of the multivariate logistic regression model, a predictive nomogram for PPCS was developed (Fig. 2). As illustrated in this nomogram, there were 12 axes in total, and axes 2–10 represented the nine variables in the predictive model. To estimate the score for each risk factor, a perpendicular line was drawn to the top points axis, and the individual scores were summed to obtain a total point value. This total point value was utilized to predict the probability of developing PPCS. For instance, consider a 77-year-old COPD patient who scored 29 points, with a preoperative leukocyte count of 3.3 × 10^9^/L (6.5 points), a PaCO_2_ level of 34.2 mmHg (35.72 points), who underwent thoracoscopic lung surgery (0 and 12 points, respectively), experienced intraoperative hypotension (21 points), had a blood loss of 55 mL (0 points), and an operation time of 71 min (0 points). The postoperative pathology report indicating a malignant tumor added another 28 points. The cumulative score for these factors was 132.22 points, corresponding to a PPCS risk probability of approximately 21%.

**Fig. 3:**
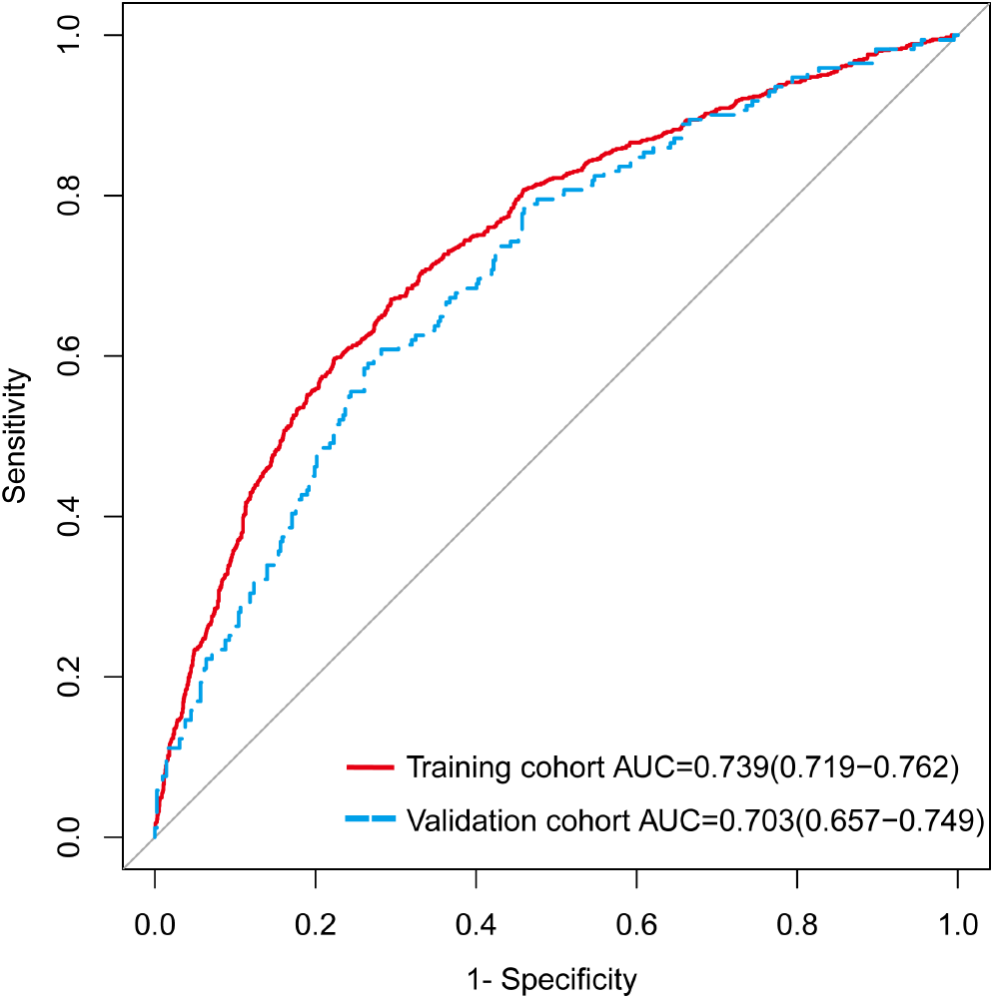
ROC curves of the nomogram for predicting PPCs in the training and validation cohorts. *Abbreviations* *ROC* receiver operating characteristic curve; *AUC* area under the ROC curve

**Fig. 4:**
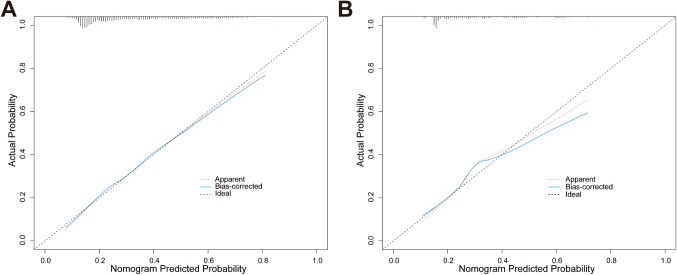
Calibration curves of the prediction nomogram in the training cohort **(A)** and validation cohort **(B)**

The discrimination capability of the predictive model and nomogram was evaluated by the ROC curve (Fig. 3), and internal validation using a 1000-bootstrap approach was conducted to reduce the optimism of the model. The area under the ROC curve (AUC) was 0.739 (95% *CI*: 0.719–0.762) for the training cohort and 0.703 (95% *CI*: 0.657–0.749) for the validation cohort, indicating the relatively acceptable prediction accuracy of the nomogram. The optimal cut-off value for the estimated probability of having a PPCS was approximately 0.367, and the sensitivity and specificity were 0.590 and 0.778, respectively. The Hosmer-Lemeshow test and calibration plot were used to assess the calibration power. The *P*-value of the Hosmer-Lemeshow test was 0.633 for the training cohort and 0.144 for the validation cohort, which suggested an insignificant difference between the predicted and observed probabilities. The correction curves of the training and validation cohorts were all almost diagonal and the calibration plots for both the training (Fig. 4A) and the validation (Fig. 4B) cohorts also demonstrated a notable calibration of the predictive nomogram.

### Clinical utility of the predictive nomogram

**Fig. 5:**
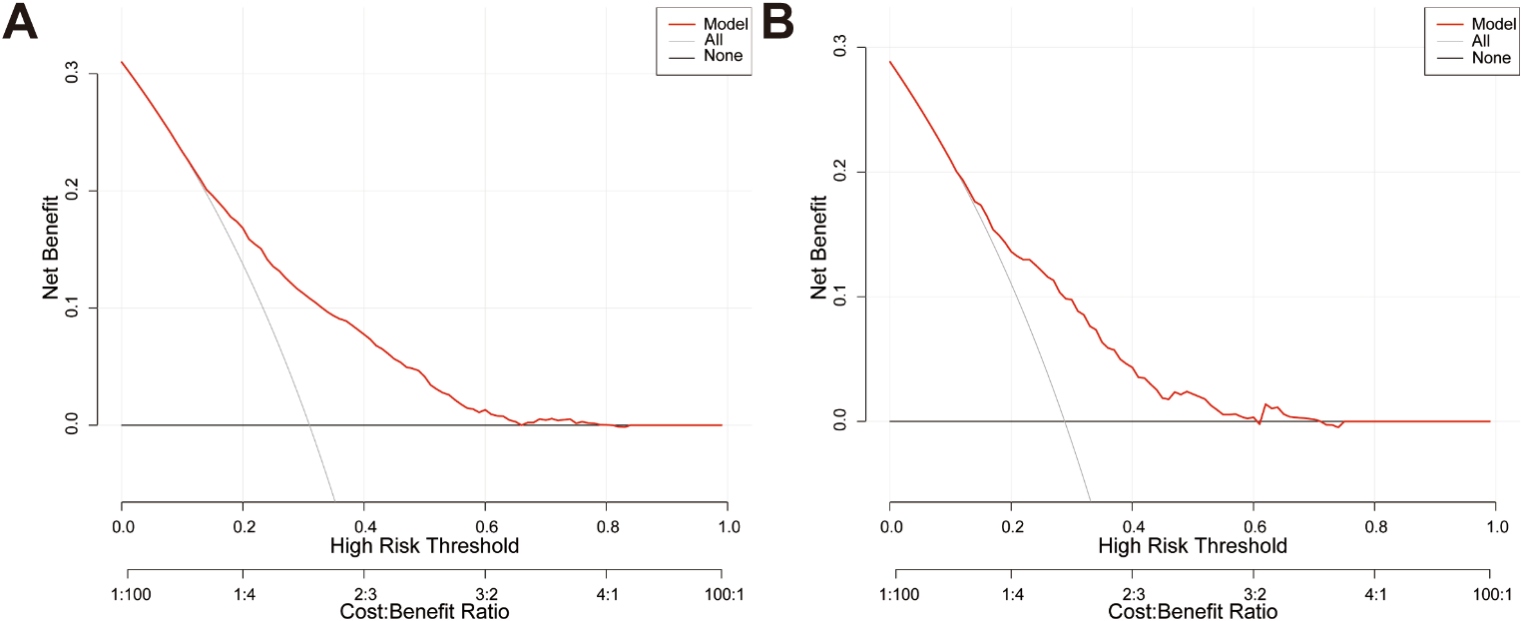
Decision curve analysis (DCA) of the prediction nomogram in the training cohort **(A)** and validation cohort **(B)**

DCA was performed to assess the clinical applicability of the predictive nomogram (Fig. 5). The DCA curve illustrated that the threshold probabilities of the prediction model in the training cohort and validation cohort were 12–84% and 8–82%, respectively. The findings indicated that the nomogram yielded a greater net benefit across a broader spectrum of threshold probabilities for predicting the risk of PPCs in both the training and validation cohorts. This finding suggested that the nomogram holds clinical relevance and has the potential to assist anesthesiologists and surgeons in making more informed clinical decisions.

### Secondary analysis of shorter inclusion period

To assess the impact of treatment guideline changes over time, secondary analysis was undertaken concentrating on the most recent 5 years of our dataset (2014 to 2019). This subset included 1,764 patients. We compared the outcomes of this cohort with those of the entire 7-year period cohort, which included 2,962 patients. It was revealed that the rates of PPCs were 15.8% in the 5-year cohort versus 16.3% in the 7-year cohort. The comparison of outcomes between the two periods did not show a statistically significant difference (*P* = 0.47), indicating that the inclusion period did not significantly influence the overall results. However, some trends were found in the incidence of pneumonia and pleural effusion, suggesting that recent advancements in treatment might positively impact these specific outcomes.

### Incorporation of year of surgery

To account for the potential impact of evolving treatment guidelines, the year of surgery was included as a variable in the multivariate analysis. The analysis demonstrated that the year of surgery was a significant predictor of PPCs, with an odds ratio of 0.94 per year (95% CI: 0.90–0.98, *P* = 0.004). This indicated that patients operated on in more recent years had lower odds of experiencing PPCs compared with those operated on in earlier years. The inclusion of the year of surgery as a covariate improved the model’s fit, as indicated by a reduction in the Akaike Information Criterion (AIC) from 820 to 805. The adjusted model exhibited a 4.5% improvement in predictive accuracy for PPCs, highlighting the importance of accounting for temporal changes in treatment protocols.

## Discussion

The incidence of postoperative pulmonary complications (PPCs) in elderly patients following thoracic surgery was found to be 31.0%. This study identified nine independent predictors of PPCs, which were utilized to develop a novel nomogram with promising predictive performance. The primary benefit of the nomogram is its ability to promptly identify high-risk patients, thereby facilitating timely interventions and improving postoperative outcomes for elderly patients undergoing thoracic surgery.

Previous studies have identified a large number of risk factors for PPCs, especially advanced age and history of undergoing thoracic surgery [4, 9, 10, 18–20]. However, there is currently no research on the risk factors for PPCs in elderly patients undergoing thoracic surgery. We identified nine independent risk factors for PPCs in elderly patients undergoing thoracic surgery were identified: preoperative presence of COPD, elevated leukocyte count, higher PaCO_2_ level, surgical site, thoracotomy, intraoperative hypotension, blood loss > 100 mL, operation time > 180 min, and malignant tumor. Some results were consistent with those of previous studies on PPCs. COPD patients are at a higher risk of PPCs due to a variety of complex and diverse physiological and anatomical reasons. The significant reduction in pulmonary function resulting from chronic bronchitis, emphysema, and other diseases limits the body’s ability to compensate after surgery, thereby increasing the risk of postoperative respiratory failure [21, 22]. Additionally, postoperative effects can exacerbate respiratory distress and contribute to respiratory muscle fatigue and failure. The chronic inflammation observed in COPD patients leads to an increase in respiratory secretions, which can result in mucus retention and potential lung infections. Moreover, the increased airway hyperresponsiveness in COPD patients can lead to bronchospasm, which is further aggravated postoperatively, causing airway obstruction and heightened respiratory distress [23].

COPD patients are at a higher risk of PPCs due to their significantly reduced pulmonary function and increased airway hyperresponsiveness [24, 25]. An elevated leukocyte count, even within the normal range, may indicate underlying conditions that require further evaluation, especially in elderly patients with declining immune function [18, 26–28]. A preoperative elevated PaCO_2_ level can lead to respiratory function impairment, increasing the risk of PPCs, particularly in COPD patients [24, 25].

Esophageal surgery, which often involves thoracotomy, presents unique challenges due to its impact on surrounding lung tissues, resulting in higher rates of PPCs [29]. Prolonged operation time and increased intraoperative bleeding are associated with various complications, such as pulmonary overinflation, respiratory muscle atrophy, postoperative anemia, and impaired tissue perfusion [30]. Intraoperative hypotension, particularly common in elderly patients, leads to poor blood circulation in the lungs, further increasing the risk of PPCs.

The presence of malignant tumors was also identified as an independent risk factor for PPCs. Tumors and their treatments can weaken the immune system, leading to greater susceptibility to PPCs. This effect is particularly pronounced in elderly patients due to their compromised immune function.

It is important to acknowledge that treatment guidelines for various procedures, including neoadjuvant therapy, RAS, Early Recovery After Surgery (ERAS), and prehabilitation, are frequently updated to reflect advancements in medical practice. These updates could potentially impact outcomes differently compared to older protocols. However, due to various limitations, not all hospitals update their treatment plans with the latest guidelines. Despite this, our study provides valuable insights into the outcomes of elderly patients undergoing thoracic surgery under the current protocols practiced in China.

### Limitations

Frailty and sarcopenia represent significant challenges in elderly patients undergoing surgery, as both conditions are associated with poor postoperative outcomes. Frailty, characterized by reduced physiological reserve and increased vulnerability to stressors, is a known predictor of adverse surgical outcomes. Sarcopenia, the loss of muscle mass and function, similarly correlates with poor recovery and increased complications. This study did not explicitly include frailty and sarcopenia as variables, which is a notable limitation. Early mobilization is a critical aspect of postoperative care, known to reduce the incidence of PPCs by enhancing respiratory function, preventing muscle atrophy, and improving overall recovery. Our study did not collect specific data on the success rate or protocol of early mobilization post-surgery. This omission is a limitation, as early mobilization practices could significantly influence the incidence of PPCs. Additionally, this study faced several other limitations: being a single-center retrospective study, the findings may not be generalizable to other settings, and retrospective data collection carries the risk of missing or misdiagnosing PPCs. There might be missing or incomplete data, particularly regarding underlying diseases and certain patient characteristics, potentially introducing bias. The leukocyte counts were within the normal range since patients scheduled for elective surgery typically have their operations postponed in cases of acute infection or elevated white blood cell count. This may limit the applicability of this indicator to broader patient populations. Future studies should incorporate assessments of frailty, such as the Frailty Index or the Clinical Frailty Scale, and sarcopenia, measured by muscle mass and function tests, to provide a more comprehensive risk profile for PPCs. Detailed documentation of early mobilization efforts, including timing, frequency, and patient compliance, should also be included to better understand its impact on PPC outcomes. Moreover, multicenter, large-sample, prospective designs are necessary to validate our findings and improve the robustness and applicability of the predictive model for PPCs in elderly thoracic surgery patients.

## Conclusions

A clinical nomogram was developed to predict the risk of PPCs in elderly patients following thoracic surgery, leveraging nine risk factors for PPCs. It demonstrated promising predictive efficacy. This nomogram can be used to estimate the risk of PPCs in elderly patients, enabling healthcare providers to implement preventive measures in advance for those identified as high-risk.

## Electronic supplementary material

Below is the link to the electronic supplementary material.

Supplementary Material 1

## Data Availability

No datasets were generated or analysed during the current study.

## Abbreviations

PPCs: Postoperative pulmonary complications
COPD: Chronic obstructive pulmonary disease
PaCO_2_: Arterial carbon dioxide
PaO_2_: Partial pressure of arterial oxygen
ASA: American Society of Anesthesiologists
BMI: Body mass index
ACEI: Angiotensin-converting enzyme inhibitor
NSAIDs: Nonsteroidal anti-inflammatory drugs
MAP: Mean artery pressure
SD: Standard deviation
IQR: Interquartile range
RAS: Robots Assisted Surgery
ERAS: Early Recovery After Surgery
TRALI: Transfusion-related acute lung injury
OR: Odds ratio
CI: Confidence interval
ROC: Receiver operating characteristic
AUC: Area under ROC curve
DCA: Decision curve analysis
