# Supplementary material for: Development and validation of a nomogram for predicting pulmonary complications in elderly patients undergoing thoracic surgery
Source: Aging Clin Exp Res. 2024 Oct 5;36(1):197. doi: 10.1007/s40520-024-02844-1 (PMC11455794; doi:10.1007/s40520-024-02844-1)
Supplement: Supplementary file 1 — Supplementary Material 1 [file 40520_2024_2844_MOESM1_ESM.docx]

**Table S1** Patient’s basic and clinical demographics between patients with and without PPCs.

| **Variable** | **Total**  **(*n* = 2962)** | **PPCs Group**  **(*n* = 918)** | **Non-PPCs Group**  **(*n* = 2044)** | ***P value*** | |
| --- | --- | --- | --- | --- | --- |
| Age (years) (IQR) | 69 (66, 72) | 69.00 (67.00, 72.00) | 69.00 (66.00, 72.00) | 0.242 | |
| Sex (male) (*n*, %) | 1807 (61.01) | 573 (62.42) | 1234 (60.37) | 0.291 | |
| BMI (kg/m^2^) (IQR) | 24.24 (22.32, 26.34) | 23.97 (22.84, 26.06) | 24.24 (22.32, 26.34) | 0.214 | |
| Smoking history (*n*, %) | 1181 (39.87) | 386 (42.05) | 795 (38.89) | 0.105 | |
| Drinking history (*n*, %) | 844 (28.49) | 284 (30.93) | 600 (29.35) | 0.384 | |
| ASA physical status |  |  |  | 0.605 | |
| I (*n*, %) | 38 (1.28) | 14 (1.53) | 24 (1.17) |  | |
| II (*n*, %) | 2505 (84.57) | 769 (83.77) | 1736 (84.93) |  | |
| III (*n*, %) | 419 (14.15) | 135 (14.71) | 284 (13.89) |  | |
| Preoperative Comorbidities |  |  |  |  | |
| Hypertension (*n*, %) | 1180 (39.84) | 351 (38.24) | 829 (40.56) | 0.232 | |
| Diabetes (*n*, %) | 620 (20.93) | 183 (19.91) | 437 (21.38) | 0.371 | |
| Coronary heart disease (*n*, %) | 327 (11.04) | 86 (9.37) | 241 (11.79) | 0.052 | |
| Arrhythmia (*n*, %) | 289 (9.76) | 86 (9.37) | 203 (9.93) | 0.681 | |
| Cerebrovascular disease (*n*, %) | 332 (11.21) | 94 (10.24) | 238 (11.64) | 0.263 | |
| COPD (*n*, %) | 547 (18.47) | 217 (23.64) | 330 (16.14) | **<0.001** | |
| Asthma (*n*, %) | 13 (0.44) | 6 (0.65) | 7 (0.34) | 0.377 | |
| Renal insufficiency (*n*, %) | 24 (0.81) | 8 (0.87) | 16 (0.78) | 0.978 | |
| Preoperative laboratory tests |  |  |  |  | |
| Leukocyte count(×10^9^/L) (IQR) | 5.95 (4.97, 7.07) | 6.13 (5.17, 7.29) | 5.88 (4.89, 6.97) | **<0.001** | |
| Hemoglobin (g/L) (IQR) | 137.56(127.34,147.81) | 136.86(128.53,145.72) | 137.58(127.95,146.39) | 0.318 | |
| PaO_2_ (mmHg) (IQR) | 96.35 (95.54, 97.45) | 96.34 (95.62, 97.39) | 96.37 (95.58, 97.48) | 0.991 | |
| PaCO_2_ (mmHg) (IQR) | 45.15 (42.33, 47.54) | 45.25 (42.45, 47.74) | 44.87 (42.22, 47.45) | **0.035** | |
| Potassium (mmol/L) (IQR) | 4.11 (3.88, 4.32) | 4.12 (3.89, 4.33) | 4.09 (3.87, 4.32) | 0.145 | |
| Sodium (mmol/L) (IQR) | 142.56(140.94,143.92) | 141.38(141.16,143.89) | 142.28(141.08,143.59) | 0.267 | |
| Glucose (mmol/L) (IQR) | 4.98 (4.59, 5.57) | 4.97 (4.64, 5.53) | 4.98 (4.59, 5.57) | 0.613 | |
| Preoperative medication |  |  |  |  | |
| ACEI drugs (*n*, %) | 90 (3.04) | 33 (3.59) | 57 (2.79) | 0.237 | |
| Statin drugs (*n*, %) | 155 (5.23) | 41 (4.47) | 114 (5.58) | 0.243 | |
| NSAIDs (*n*, %) | 2679 (90.45) | 818 (89.11) | 1861 (91.05) | 0.097 | |
| β -blocker (*n*, %) | 257 (8.68) | 84 (9.15) | 173 (8.46) | 0.539 | |
| Calcium channel blocker (*n*, %) | 698 (23.57) | 205 (22.33) | 493 (24.12) | 0.289 | |
| Preoperative heart rate (IQR) | 74 (69, 78) | 74 (69, 78) | 73 (68, 78) | 0.091 | |
| Preoperative MAP (mmHg) (SD) | 95.55 (10.97) | 94.33 (10.93) | 96.18 (10.94) | **<0.001** | |
| *Abbreviations*: *BMI*: body mass index; *ASA*: American Society of Anesthesiologists; *COPD*: chronic obstructive pulmonary disease; *PaO_2_*: arterial partial pressure of oxygen; *PaCO_2_*: arterial partial pressure of carbon dioxide; *ACEI*: angiotensin-converting enzyme inhibitors; *NSAIDs*: non-steroidal anti-inflammatory drugs; *MAP*: mean artery pressure; *IQR*: interquartile range; *SD*: standard deviation. | | | | |  |
